# Supplementary material for: Higher Infection Risk among Health Care Workers and Lower Risk among Smokers Persistent across SARS-CoV-2 Waves—Longitudinal Results from the Population-Based TiKoCo Seroprevalence Study
Source: Int J Environ Res Public Health. 2022 Dec 17;19(24):16996. doi: 10.3390/ijerph192416996 (PMC9779618; doi:10.3390/ijerph192416996)

Supplementary Table S1. Participant characteristics at baseline and follow-up. For the participants at baseline (BL) and participants as well as dropouts among the two follow-ups (FU1, FU2), descriptive statistics are shown by characteristics (median, minimum, maximum and interquartile range, IQR, or proportions and absolute numbers) and [number of individuals with valid information]. We also provide p-values of Kruskal-Wallis tests (for categorical/binary variables) or Chi-squared tests (for quantitative variables) comparing the participants and dropouts per Follow-up (at FU1 and at FU2). Fraction participated provides for each subgroup of (BL) participants, the fraction of participants at follow-up (FU1, FU2; not given for quantitative variables). All characteristics are self-reported at baseline, except N-seropositivity which stems from blood draw at baseline (i.e., proportion of positives for N-specific antibodies, cut-off=1.0).

	BL	Follow-up 1 (FU1)			Follow-up 2 (FU2)			Fraction participated	
	**Participants**	**Participants**	**Dropouts**	**P-Val.**	**Participants**	**Dropouts**	**P-Val.**	**FU1**	**FU2**
**Age, Sex (BL)**	**[n=4181]**	**[n=3513]**	**[n=668]**		**[n=3374]**	**[n=807]**		**0.84**	**0.81**
Median age (min, max, IQR)	52.0 (14.0, 102.0, 35.0-64.0)	53.0 (14.0, 102.0, 37.0-64.0)	42.0 (14.0, 93.0, 29.0-58.0)	<0.001	53.0 (14.0, 102.0, 37.0-64.0)	44.0 (14.0, 91.0, 30.0-59.0)	<0.001	-	-
14-19: % (#)	5.4 (225)	5.0 (176)	7.3 (49)	0.019	5.2 (177)	5.9 (48)	0.48	0.78	0.79
20-49: % (#)	40.8 (1707)	38.3 (1345)	54.2 (362)	<0.001	38.1 (1284)	52.4 (423)	<0.001	0.79	0.75
50-69: % (#)	38.8 (1624)	41.2 (1449)	26.2 (175)	<0.001	41.2 (1389)	29.1 (235)	<0.001	0.89	0.86
70+: % (#)	14.9 (625)	15.5 (543)	12.3 (82)	0.04	15.5 (524)	12.5 (101)	0.035	0.87	0.84
Women: % (#)	51.6 (2158)	53.0 (1861)	44.5 (297)	<0.001	53.7 (1813)	42.8 (345)	<0.001	0.86	0.84
**Chronic diseases:**	**[n=4081]**	**[n=3435]**	**[n=646]**		**[n=3300]**	**[n=781]**			
Autoimmune: % (#)	7.1 (289)	7.3 (250)	6.0 (39)	0.296	7.4 (243)	5.9 (46)	0.172	0.87	0.84
Cancer: % (#)	4.9 (202)	5.2 (178)	3.7 (24)	0.139	5.0 (164)	4.9 (38)	0.977	0.88	0.81
Type-2 diab.: % (#)	7.6 (312)	7.5 (259)	8.2 (53)	0.615	7.4 (245)	8.6 (67)	0.309	0.83	0.79
Cardiovasc.: % (#)	9.9 (402)	9.6 (331)	11.0 (71)	0.323	9.5 (314)	11.3 (88)	0.158	0.82	0.78
None of these: % (#)	75.8 (3093)	75.6 (2596)	76.9 (497)	0.49	76.0 (2507)	75.0 (586)	0.614	0.84	0.81
**Education**	**[n=4085]**	**[n=3433]**	**[n=652]**		**[n=3301]**	**[n=784]**			
Years: median (min, max, IQR)	11.0 (6.0, 22.0, 10.0-14.0)	11.0 (6.0, 22.0, 10.0-13.0)	11.0 (6.0, 21.0, 10.0-14.0)	0.406	11.0 (6.0, 22.0, 10.0-14.0)	11.0 (6.0, 21.0, 10.0-14.0)	0.365	-	-
>=13 years: % (#)	30.0 (1225)	29.5 (1013)	32.5 (212)	0.136	29.8 (985)	30.6 (240)	0.703	0.83	0.80
**Occupation (BL)**	**[n=3303]**	**[n=2773]**	**[n=530]**		**[n=2652]**	**[n=651]**			
Curr. working: % (#)	74.0 (2444)	73.8 (2046)	75.1 (398)	0.564	74.1 (1965)	73.6 (479)	0.827	0.84	0.80
Medical: % (#)	8.0 (263)	8.1 (224)	7.4 (39)	0.636	8.1 (216)	7.2 (47)	0.484	0.85	0.82
Education: % (#)	3.7 (121)	3.6 (101)	3.8 (20)	0.983	3.6 (95)	4.0 (26)	0.701	0.83	0.79
Grocery: % (#)	3.1 (104)	3.2 (90)	2.6 (14)	0.553	3.3 (87)	2.6 (17)	0.453	0.87	0.84
**Smoking**	**[n=4157]**	**[n=3493]**	**[n=664]**		**[n=3356]**	**[n=801]**			
Never smoking: % (#)	54.9 (2282)	56.7 (1981)	45.3 (301)	<0.001	57.3 (1923)	44.8 (359)	<0.001	0.87	0.84
Ex-smoker: % (#)	24.7 (1025)	24.6 (860)	24.8 (165)	0.939	24.6 (827)	24.7 (198)	1	0.84	0.81
Current smoker: % (#)	20.4 (850)	18.7 (652)	29.8 (198)	<0.001	18.1 (606)	30.5 (244)	<0.001	0.77	0.71

**Alcohol**	**[n=4049]**	**[n=3412]**	**[n=637]**		**[n=3280]**	**[n=769]**			
Alc. drinks, daily: median (min, max, IQR)	0.2 (0.0, 8.0, 0.0-0.6)	0.2 (0.0, 8.0, 0.0-0.6)	0.2 (0.0, 5.5, 0.0-0.6)	0.006	0.2 (0.0, 8.0, 0.0-0.6)	0.2 (0.0, 8.0, 0.0-0.6)	0.196	-	-
>2 alc. drinks, daily: % (#)	6.5 (262)	6.3 (216)	7.2 (46)	0.453	6.2 (204)	7.5 (58)	0.207	0.82	0.78
**Other lifestyle factors**									
BMI: median (min, max, IQR)	26.6 (13.9, 62.1, 23.7-30.4) [n=4134]	26.6 (13.9, 62.1, 23.7-30.3) [n=3474]	26.8 (16.0, 56.5, 23.9-30.4) [n=660]	0.396	26.6 (13.9, 62.1, 23.7-30.4) [n=3339]	26.3 (16.0, 56.5, 23.7-30.1) [n=795]	0.556	NA	NA
**N-specific antibodies**									
% (#)	8.9 (374) [n=4181]	10.0 (351) [n=3513]	3.4 (23) [n=668]	<0.001	10.3 (349) [n=3374]	3.1 (25) [n=807]	<0.001	0.94	0.93
% (#), age 14-19 yrs	10.7 (24) [n=225]	13.6 (24) [n=176]	0.0 (0) [n=49]	0.013	13.0 (23) [n=177]	2.1 (1) [n=48]	0.056	1.00	0.96
% (#), age 20-49 yrs	8.6 (146) [n=1707]	10.0 (135) [n=1345]	3.0 (11) [n=362]	<0.001	10.4 (133) [n=1284]	3.1 (13) [n=423]	<0.001	0.92	0.91
% (#), age 50-69 yrs	9.3 (151) [n=1624]	9.8 (142) [n=1449]	5.1 (9) [n=175]	0.062	10.3 (143) [n=1389]	3.4 (8) [n=235]	0.001	0.94	0.95
% (#), age 70+ yrs	8.5 (53) [n=625]	9.2 (50) [n=543]	3.7 (3) [n=82]	0.142	9.5 (50) [n=524]	3.0 (3) [n=101]	0.048	0.94	0.94
% (#), never-smoker	9.9 (226) [n=2282]	10.8 (213) [n=1981]	4.3 (13) [n=301]	<0.001	11.0 (212) [n=1923]	3.9 (14) [n=359]	<0.001	0.94	0.94
% (#), ex-smoker	10.3 (106) [n=1025]	11.4 (98) [n=860]	4.8 (8) [n=165]	0.017	11.7 (97) [n=827]	4.5 (9) [n=198]	0.004	0.92	0.92
% (#) current smoker	4.9 (42) [n=850]	6.1 (40) [n=652]	1.0 (2) [n=198]	0.006	6.6 (40) [n=606]	0.8 (2) [n=244]	<0.001	0.95	0.95

Supplementary Table S2. Age- and sex-adjusted odds ratios for new seropositivity between smoking and occupational groups at three study time points (BL, between BL and FU1, and between FU1 and FU2). Shown are absolute numbers of individuals at risk, number of newly sero-positives (based on N-specific antibodies), the corresponding fraction of newly sero-positives in the subgroups and age-sex adjusted Odds ratios as well as 95%-confidence intervals and p-Values compared to a reference category (never smokers, and “other” occupations, respectively). Analysis regarding occupation are restricted to individuals aged 20-69 years and we refrain from providing odds ratio estimates for groups with zero events (new seropositives).

			BL				
Smoking	# at risk	# new sero-pos.	Fraction sero-pos	OR	2.5%-CI	97.5%-CI	P-Val.
Current	850	42	0.049	0.47	0.33	0.66	$1.3*10^{-5}$
Ex	1025	106	0.103	1.07	0.83	1.38	0.59
Never	2282	226	0.099	Ref.	-	-	-
Occupation	# at risk	# new sero-pos.	Fraction sero-pos	OR	2.5%-CI	97.5%-CI	P-Val.
Teacher	121	11	0.091	1.10	0.57	2.12	0.77
Grocery	104	7	0.067	0.77	0.34	1.71	0.51
Medical	263	41	0.156	1.99	1.36	2.93	$3.3*10^{-4}$
other	2815	235	0.083	Ref.	-	-	-
			FU1				
Smoking	# at risk	# new sero-pos.	Fraction sero-pos	OR	2.5%-CI	97.5%-CI	P-Val.
Current	607	2	0.003	0.40	0.09	1.81	0.22
Ex	827	5	0.006	0.73	0.25	2.12	0.56
Never	1705	14	0.008	Ref.	-	-	-
Occupation	# at risk	# new sero-pos.	Fraction sero-pos	OR	2.5%-CI	97.5%-CI	P-Val.
Teacher	91	0	0	-	-	-	-
Grocery	83	0	0	-	-	-	-
Medical	186	2	0.011	1.41	0.29	6.80	0.67
other	2139	13	0.006	Ref.	-	-	-
			FU2				
Smoking	# at risk	# new sero-pos.	Fraction sero-pos	OR	2.5%-CI	97.5%-CI	P-Val.
Current	520	19	0.037	0.56	0.33	0.94	0.02
Ex	763	49	0.064	1.21	0.83	1.78	0.31
Never	1517	94	0.062	Ref.	-	-	-
Occupation	# at risk	# new sero-pos.	Fraction sero-pos	OR	2.5%-CI	97.5%-CI	P-Val.
Teacher	77	5	0.065	1.19	0.46	3.11	0.72
Grocery	76	7	0.092	1.87	0.81	4.27	0.13
Medical	166	25	0.151	3.17	1.92	5.24	$4.48*10^{-6}$
other	1906	99	0.052	Ref.	-	-	-

Supplementary Table S3. Age- and sex-adjusted odds ratios for cross-sectional seropositivity between smoking and occupational groups at FU2 based on N-binding and S-binding antibodies. Shown are absolute numbers of individuals at risk, number of sero-positives, the corresponding fraction of sero-positives and age-sex adjusted odds ratios as well as 95%-confidence intervals and p-Values compared to a reference category (never smokers, and “other” occupations, respectively). Analyses are restricted to unvaccinated individuals and analyses regarding occupation are restricted to individuals aged 20-69. Note, that these cross-sectional association estimates can be biased towards too strong associations when receiving a positive antibody test result from the BL/FU1 test increases probability of continued participation in the later follow-ups of the study.

N-antibodies								S-antibodies							
Smoking	# at risk	# sero-pos.	Fraction sero-pos.	OR	2.5%-CI	97.5%-CI	P-Val.	Smoking	# at risk	# sero-pos.	Fraction sero-pos.	OR	2.5%-CI	97.5%-CI	P-Val.
never	1021	229	0.22	Ref.	-	-	-	never	1020	248	0.24	Ref.	-	-	-
ex	444	104	0.23	1.09	0.83	1.45	0.53	ex	444	111	0.25	1.07	0.81	1.41	0.61
current	344	41	0.12	0.47	0.32	0.68	$4.18*10^{-5}$	current	344	46	0.13	0.49	0.34	0.70	$5.42*10^{-5}$
Occupation	# at risk	# sero-pos.	Fraction sero-pos	OR	2.5%-CI	97.5%-CI	P-Val.	Occupation	# at risk	# sero-pos.	Fraction sero-pos	OR	2.5%-CI	97.5%-CI	P-Val.
other	1453	258	0.18	Ref.	-	-	-	other	1453	283	0.19	Ref.	-	-	-
teacher	39	10	0.26	1.75	0.82	3.75	0.14	teacher	39	10	0.26	1.57	0.73	3.35	0.24
grocery	63	12	0.19	1.12	0.57	2.18	0.74	grocery	63	13	0.21	1.12	0.59	2.13	0.73
medical	71	38	0.54	5.76	3.43	9.66	$1.28*10^{-11}$	medical	71	38	0.54	5.21	3.12	8.71	$1.35*10^{-10}$

Supplementary Table S4. Association of current smoking and new sero-positivity at BL and FU2 in subgroups. Shown are absolute numbers of individuals at risk, number of newly sero-positives (based on N-specific antibodies), the corresponding fraction of newly sero-positives and raw odds ratios for new seropositivity between current- and never smokers (and 95% confidence intervals) in multiple subgroups of the study participants: age >=/<50 years, sex: women/men, Education: >=/<13 years, chronic disease (autoimmune, cancer, type-2 diabetes, cardiovascular diseases) and none of these, medical occupation yes/no (including all other occupations and unemployed).

	Baseline				Follow-up 2			
Subgroup	# at risk (smoker/never-smoker)	# new sero-positive (smoker/never-smoker)	% new sero-positive (smoker/never-smoker)	OR (95%-CI)	# at risk (smoker/never-smoker)	# new sero-positive (smoker/never-smoker)	% new sero-positive (smoker/never-smoker)	OR (95%-CI)
Age: <50	464/1103	23/117	5.0%/10.6%	0.44 (0.28-0.70)	235/701	13/55	5.5%/7.8%	0.69 (0.37-1.28)
Age: 50+	386/1179	19/109	4.9%/9.2%	0.51 (0.31-0.84)	285/816	6/39	2.1%/4.8%	0.43 (0.18-1.02)
Sex: men	441/981	17/100	3.9%/10.2%	0.35 (0.21-0.60)	255/617	9/45	3.5%/7.3%	0.47 (0.22-0.97)
Sex: women	409/1301	25/126	6.1%/9.7%	0.61 (0.39-0.95)	265/900	10/49	3.8%/5.4%	0.68 (0.34-1.36)
Education: high	194/745	3/73	1.5%/9.8%	0.14 (0.05-0.46)	123/482	5/28	4.1%/5.8%	0.69 (0.26-1.82)
Education: low	636/1487	39/146	6.1%/9.8%	0.60 (0.42-0.87)	384/1007	14/65	3.6%/6.5%	0.55 (0.30-0.99)
Chronic disease	177/481	11/47	6.2%/9.8%	0.61 (0.31-1.21)	116/305	3/13	2.6%/4.3%	0.60 (0.17-2.13)
No chronic disease	646/1755	30/175	4.6%/10.0%	0.44 (0.30-0.65)	392/1184	15/78	3.8%/6.6%	0.56 (0.32-0.99)
Medical occupation: yes	79/124	9/23	11.4%/18.5%	0.56 (0.25-1.29)	43/73	4/10	9.3%/13.7%	0.65 (0.19-2.20)
Medical ocupation: no	765/2130	32/202	4.2%/9.5%	0.42 (0.28-0.61)	474/1429	15/84	3.2%/5.9%	0.52 (0.30-0.92)

Supplementary Table S5. Age- and sex-adjusted odds ratios for a positive test between smoking and occupational groups at three study time points (BL, between BL and FU1, and between FU1 and FU2). Analyses are restricted to all individuals that reported being tested in the respective study period (self-report). Shown are absolute numbers of tested individuals, number of positive tests (health authority validated), the corresponding fraction of positive tests in the subgroups and age-sex adjusted odds ratios as well as 95%-confidence intervals and p-Values compared to a reference category (never smokers, and "other" occupations, respectively). Analyses regarding occupation are restricted to individuals aged 20-69 years and we refrain from providing odds ratio estimates for groups with zero events (positive tests).

			BL				
Smoking	# tested	# pos. Test	Fraction pos. Test	OR	2.5%-CI	97.5%-CI	P-Val.
Current	105	7	0.067	0.36	0.15	0.85	0.018
Ex	148	24	0.162	0.76	0.42	1.35	0.337
Never	248	43	0.173	Ref.	-	-	-
Occupation	# tested	# pos. Test	Fraction pos. Test	OR	2.5%-CI	97.5%-CI	P-Val.
Teacher	30	3	0.100	0.80	0.22	2.91	0.730
Grocery	5	0	0.000	-	-	-	-
Medical	116	14	0.121	1.09	0.52	2.27	0.813
other	257	38	0.148	Ref.	-	-	-
			FU1				
Smoking	# tested	# pos. Test	Fraction pos. Test	OR	2.5%-CI	97.5%-CI	P-Val.
Current	200	1	0.005	0.22	0.03	1.81	0.151
Ex	252	7	0.028	1.11	0.41	2.99	0.840
Never	493	13	0.026	Ref.	-	-	-
Occupation	# tested	# pos. Test	Fraction pos. Test	OR	2.5%-CI	97.5%-CI	P-Val.
Teacher	60	1	0.017	0.79	0.09	6.58	0.821
Grocery	19	0	0.000	-	-	-	-
Medical	127	2	0.016	0.73	0.15	3.59	0.696
other	606	12	0.020	Ref.	-	-	-
			FU2				
Smoking	# tested	# pos. Test	Fraction pos. Test	OR	2.5%-CI	97.5%-CI	P-Val.
Current	285	18	0.063	0.58	0.33	1.00	0.045
Ex	418	49	0.117	1.28	0.85	1.91	0.225
Never	737	77	0.104	Ref.	-	-	-
Occupation	# tested	# pos. Test	Fraction pos. Test	OR	2.5%-CI	97.5%-CI	P-Val.
Teacher	63	6	0.095	1.10	0.45	2.71	0.824
Grocery	31	6	0.194	2.58	1.00	6.65	0.046
Medical	175	22	0.126	1.52	0.89	2.59	0.118
other	1027	90	0.088	Ref.	-	-	-

Supplementary Figure S1. Vaccination status at FU2 among different subgroups of study participants. Shown are the fractions of fully vaccinated individuals (two vaccinations by Comirnaty (BionNTech), SpikeVax (Moderna), or Vaxzevria (AstraZeneca) ≥14 days before FU2 assessment), partly vaccinated (at least one vaccination but not fully vaccinated), and unvaccinated individuals, as well as individuals without information on vaccination status, for different age and occupation subgroups (Panel A) as well as smoking status (Panel B).

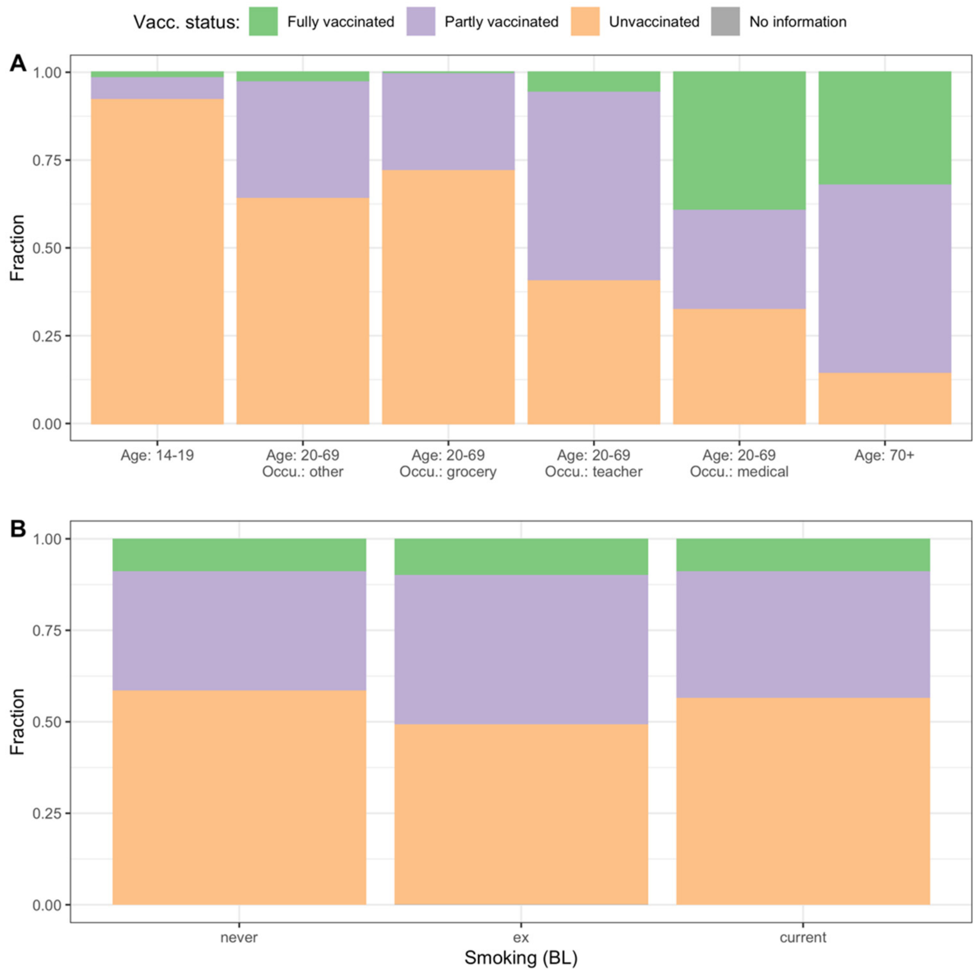

Supplementary Figure S2: Dose-response association of smoking with new seropositivity at BL and FU2. Show is the model-based probability of new seropositivity (N-based) in association with the daily number of cigarettes (self-reported, winsorized at 30) and the associated 95% confidence interval. The association was estimated using a generalized additive model allowing for a (potentially) non-linear association of the log-odds of new seropositivity with the number of smoked cigarettes. The model was estimated separately for the BL and FU2 assessment. We observe a decrease in the probability of new seropositivity with an increasing number of smoked cigarettes. Note that the overall level of the model-based probability at BL and FU2 differs because of fewer new seropositive cases between FU1 and FU2 compared to the number of new seropositive cases before the BL assessment (cf. Fig. 1 in the main text).

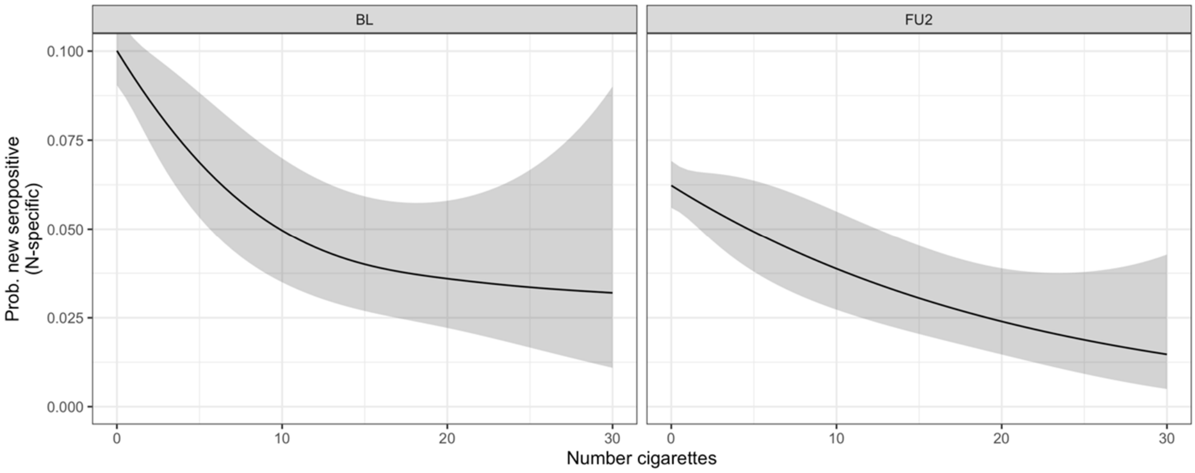

Supplementary Figure S3 (A): Association estimates from full model. We estimated multiple logistic regression to analyze the association of (new) seropositivity with medical occupation, and current smoking when accounting for sociodemographic as well as lifestyle factors (age, sex, education, household size, physical activity, alcohol consumption, and BMI) at BL and FU2 (vs. FU1). This analysis was not done for FU1 due to few newly infected individuals in this observation period. We show estimated odds-ratios and 95% confidence intervals for the respective category compared to the [reference category]. Supplementary Figure 1B shows the results restricted to the population aged 20-69 (restricted to age-groups that are usually in employment/adults). **(B) Association estimates from full model; analysis restricted to adults (aged 20-69).**

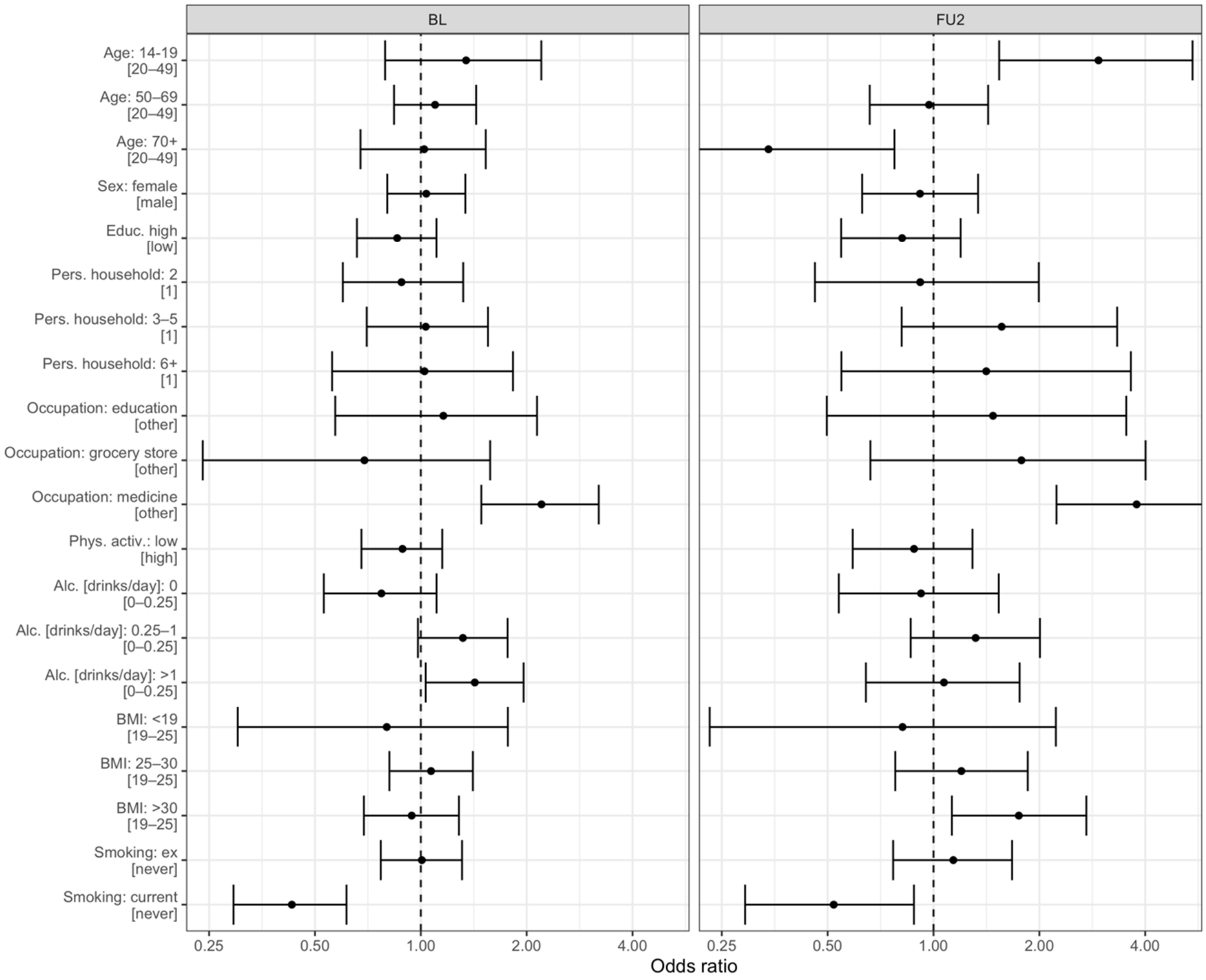

(A)

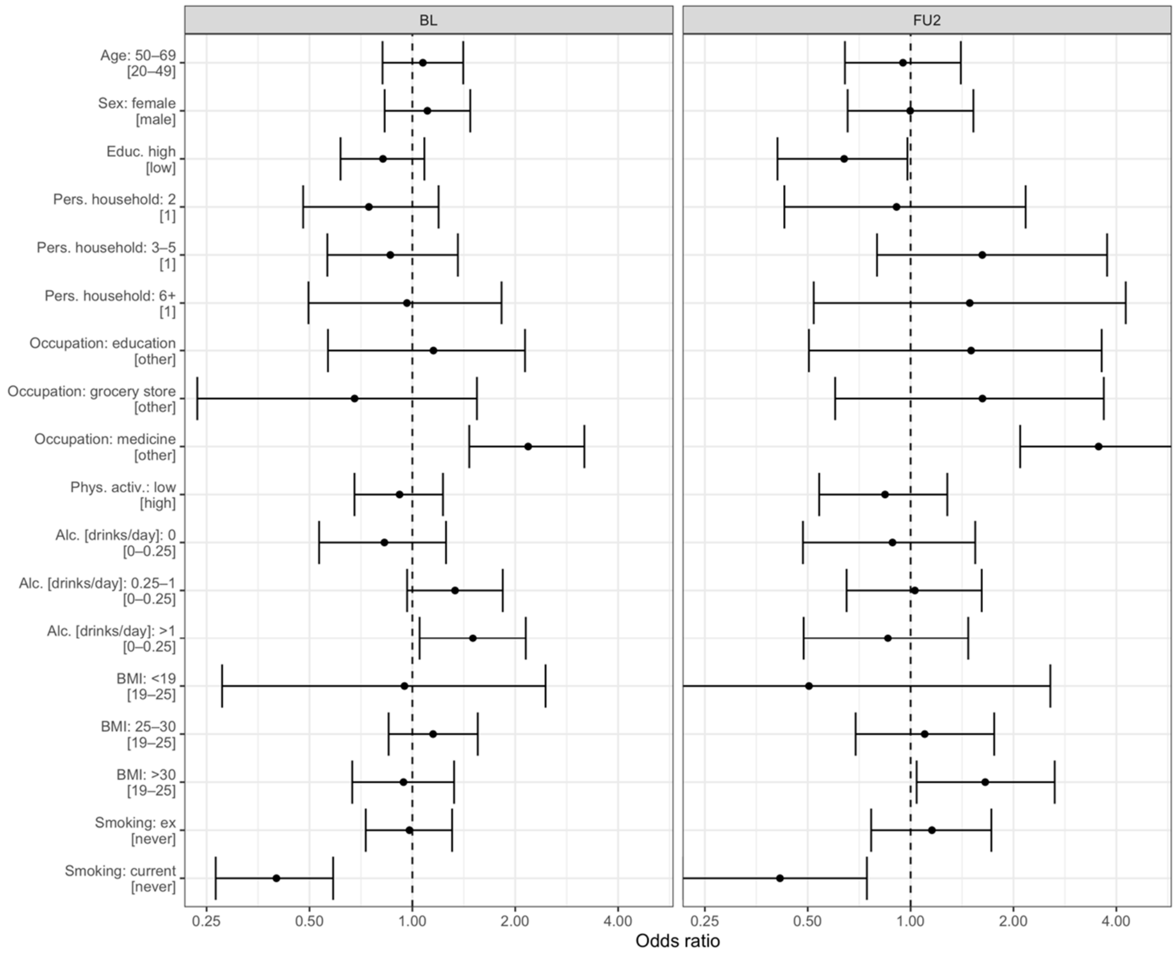
BL
FU2
Age: 50–69
[20–49]
Sex: female
[male]
Educ. high
[low]
Pers. household: 2
[1]
Pers. household: 3–5
[1]
Pers. household: 6+
[1]
Occupation: education
[other]
Occupation: grocery store
[other]
Occupation: medicine
[other]
Phys. activ.: low
[high]
Alc. [drinks/day]: 0
[0–0.25]
Alc. [drinks/day]: 0.25–1
[0–0.25]
Alc. [drinks/day]: >1
[0–0.25]
BMI: <19
[19–25]
BMI: 25–30
[19–25]
BMI: >30
[19–25]
Smoking: ex
[never]
Smoking: current
[never]
0.25
0.50
1.00
2.00
4.00
Odds ratio

(B)

Supplementary Figure S4: Association of current smoking and new seropositivity in subgroups. Shown are the proportions of new seropositives, number of seropositives, and number at risk at BL (June 2020) and FU1 to FU2 (Nov 2020 - April 2021) for never and current smokers among different subgroups oft he study participatns: BL age <50 and >= 50 years, sex: male and female, education high (>=13 years) and low, chronic diseases (cancer, autoimmune, type-2 diabetes, cardiovascular diseases) and none of theses, as well as medical occupation (yes/no, including unemployed). Whiskers represent 95% confidence intervals.

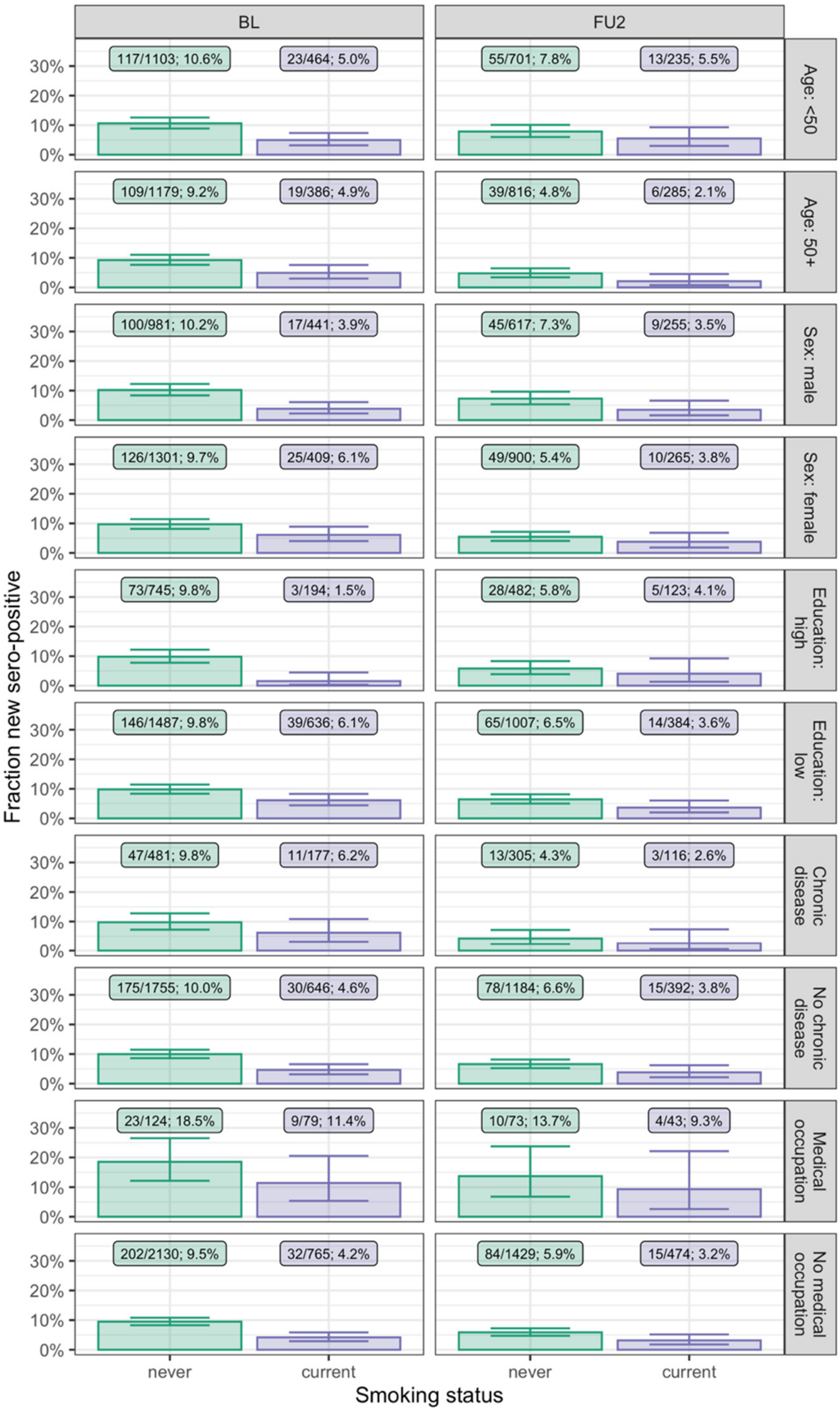

Supplement: Supplementary file 1 [file ijerph-19-16996-s001.zip › ijerph-2018939-supplementary.pdf]
